# Clinically feasible biomechanical assessment of lower limb strength and postural stability in adults aged 50–75 years with moderate knee osteoarthritis: implications for technology-enabled healthy aging

**DOI:** 10.3389/fbioe.2026.1797065

**Published:** 2026-05-28

**Authors:** Adel Alshahrani, Ravi Shankar Reddy

**Affiliations:** 1 Physical Therapy Program, Department of Medical Rehabilitation Sciences, College of Applied Medical Sciences, Najran University, Najran, Saudi Arabia; 2 Health Research Center, Najran University, Najran, Saudi Arabia; 3 Program of Physical Therapy, Department of Medical Rehabilitation Sciences, College of Applied Medical Sciences, King Khalid University, Abha, Saudi Arabia

**Keywords:** biomechanics, dynamometry, knee osteoarthritis, muscle strength, postural sway, posturography

## Abstract

**Objectives:**

This study aimed to compare lower-limb muscle strength and postural sway between individuals with KOA and healthy controls, and to examine the association between strength parameters and postural stability using clinically feasible assessment methods.

**Methods:**

A cross-sectional analysis was conducted among 90 participants (45 KOA, 45 matched controls). The participants were older adults aged 50–75 years, and 40.0% of the KOA group and 42.22% of the control group were male. Participants with knee osteoarthritis were classified as having moderate disease severity based on Kellgren–Lawrence grades II–III. Strength and balance assessments were conducted in a counterbalanced order, with a standardized 10-min rest interval between testing sessions to minimize fatigue effects. Isometric quadriceps and hamstring strength were measured using handheld dynamometry. Postural sway metrics—sway area, sway velocity, and single-leg stance time—were assessed via static posturography. Limb symmetry index (LSI) and quadriceps-to-hamstrings (Q: H) ratio were calculated. Pearson correlation and multiple linear regression analyses examined associations between strength and postural control.

**Results:**

KOA participants showed significantly greater sway area and velocity, reduced stance time, and lower quadriceps and hamstrings strength compared to controls (all p < 0.001). Quadriceps strength (β = −0.024, p < 0.001) and LSI (β = −0.062, p = 0.001) were independent predictors of sway area under eyes-closed conditions. LSI and quadriceps strength were strongly correlated with sway parameters and stance performance.

**Conclusion:**

Lower limb strength deficits and inter-limb asymmetry significantly contribute to postural instability in individuals with KOA. Objective, clinically feasible tools such as handheld dynamometry and posturography can support evaluation and inform rehabilitation strategies targeting strength and balance.

## Introduction

Knee osteoarthritis (KOA) is a prevalent degenerative joint disorder characterized by progressive cartilage degradation, joint space narrowing, and osteophyte formation (Lv et al., 2021). It significantly impairs mobility and quality of life, particularly among older adults, and is a leading contributor to physical disability worldwide (Atukorala and Hunter, 2023). Beyond pain and joint stiffness, KOA is associated with impaired neuromuscular function, reduced proprioceptive acuity, and compromised postural control (Zeng et al., 2022). These sensorimotor deficits contribute to increased fall risk and functional dependency, particularly in activities requiring balance and dynamic postural adjustments (Shen et al., 2024; Alshahrani et al., 2025; Kandakurti et al., 2025; Reddy et al., 2022; Ahmad et al., 2023). As such, accurate assessment of balance and muscle function is essential for effective clinical management and fall prevention in individuals with KOA (Hendawy Saad, 2025).

Previous studies have demonstrated that individuals with KOA exhibit greater postural sway and reduced static and dynamic balance compared to healthy adults (Zeng et al., 2022; Sarvestani et al., 2025; Ucurum et al., 2024). Zeng et al. (2022) reported increased postural instability in KOA patients, which they associated with proprioceptive decline and muscle weakness. Similarly, Sarvestani et al. (2025). observed altered sensorimotor performance and reduced stance stability in KOA, particularly during single-limb tasks. Hislop et al. (2022) and Ucurum et al. (2024) further highlighted the relationship between lower-limb muscle strength, particularly quadriceps force, and balance performance, linking strength deficits to increased sway and impaired postural control. These findings underscore the biomechanical interdependence between muscle strength and balance function in individuals with KOA (Zeng et al., 2022). Despite the established relevance of these parameters, many prior investigations have relied on either complex laboratory-based equipment or self-reported measures, thereby limiting their direct applicability to routine clinical practice.

There remains a critical need to quantify the relationship between lower limb muscle strength and postural stability in KOA using objective, accessible, and clinically feasible tools. While the individual effects of quadriceps weakness and balance impairment have been described, few studies have systematically examined their interrelationship using device-based strength testing and quantitative posturography in tandem (Hendawy Saad, 2025; Hendawy, 2023). Furthermore, parameters such as inter-limb strength symmetry and the quadriceps-to-hamstrings (Q: H) ratio have received limited attention, despite their potential contributions to postural control (Costantino et al., 2025). Understanding these associations is essential for refining assessment strategies and guiding targeted interventions, particularly in rehabilitation settings where precision in identifying functional deficits can enhance treatment outcomes (Costantino et al., 2025).

The objective of this study was to compare postural sway and lower-limb muscle strength between individuals with KOA and healthy controls, and to examine the associations between strength variables and postural stability. It was hypothesized that individuals with KOA would demonstrate significantly impaired postural control and lower muscle strength than controls, and that quadriceps strength, Q: H ratio, and limb symmetry would significantly predict postural sway performance.

## Materials and methods

### Study design, ethics, and settings

This cross-sectional observational study was conducted from 12/05/2024 to 10/04/2025 at the Biomechanics Lab, Musculoskeletal Rehabilitation Clinic, Applied Medical Sciences College, in Abha City, Saudi Arabia. Ethical approval was obtained from the Institutional Review Board of King Khalid University (KKU-163-2025-31), and written informed consent was obtained from all participants before enrollment. The study was conducted in full compliance with the ethical standards of the Declaration of Helsinki.

### Participants

Participants were recruited through consecutive sampling from the Musculoskeletal Rehabilitation Clinic’s outpatient referrals at the University. All individuals referred for assessment and management of knee pain were initially screened for eligibility by a licensed physical therapist with clinical expertise in musculoskeletal disorders. The diagnosis of knee osteoarthritis was confirmed based on the American College of Rheumatology (ACR) clinical and radiographic criteria (Joo et al., 2022; Lee et al., 2021), which include the presence of knee pain accompanied by at least one of the following: age over 50 years, morning stiffness lasting less than 30 min, crepitus on active motion, and radiographic evidence consistent with Kellgren–Lawrence grades II or III (Macri et al., 2022; Alhasan et al., 2025). Knee radiographs were evaluated by a musculoskeletal radiologist blinded to group allocation using the Kellgren–Lawrence grading system. Only grades II and III were included to reflect moderate OA severity.

Inclusion criteria for the KOA group were adults aged 50–75 years, unilateral or bilateral knee osteoarthritis of at least 6 months’ duration, ability to ambulate independently without assistive devices over short distances, and stable medication use for at least 4 weeks. Participants in the control group were age- and sex-matched individuals without any clinical or radiographic evidence of knee pathology, recruited from community advertisements and general health screening programs. Exclusion criteria for all participants included a history of lower limb surgery within the past year, diagnosed neurological or vestibular disorders affecting balance, uncontrolled cardiovascular conditions, significant cognitive impairment, or any other musculoskeletal conditions that could interfere with strength or balance assessments. Participants with uncorrected visual impairment or clinically significant visual deficits that could affect postural control or the ability to perform balance assessments were also excluded to minimize potential confounding effects related to visual input. Eligibility was confirmed through a structured clinical interview and, when applicable, radiograph review. Baseline demographic and clinical data were collected after eligibility confirmation and before participation in strength and balance assessments. The final sample consisted of 90 participants, including 45 individuals with knee osteoarthritis (KOA) and 45 age- and sex-matched controls (Table 1). Participants with KOA and controls were similar in age (65.42 ± 5.88 vs. 64.73 ± 6.11 years) and sex distribution, with no significant difference in limb dominance. However, the KOA group had a higher body mass index (29.76 ± 3.45 vs. 27.83 ± 3.11 kg/m^2^), lower physical activity levels, and a greater proportion of assistive device use. Falls in the past year were more frequent in the KOA group. The mean duration of KOA was 5.38 ± 2.14 years, and 62.22% were classified as Kellgren–Lawrence grade II.

**TABLE 1:** Demographic and clinical characteristics of participants.

Variable	KOA group (n = 45)	Control group (n = 45)	p-value
Age (years), mean ± SD	65.42 ± 5.88	64.73 ± 6.11	0.541
Sex (male/Female), n (%)	18 (40.00%)/27 (60.00%)	19 (42.22%)/26 (57.78%)	0.827
BMI (kg/m^2^), mean ± SD	29.76 ± 3.45	27.83 ± 3.11	0.009
Duration of KOA (years), mean ± SD	5.38 ± 2.14	—	—
Kellgren–Lawrence grade II/III, n (%)	28 (62.22%)/17 (37.78%)	—	—
Dominant leg (right/Left), n (%)	41 (91.11%)/4 (8.89%)	39 (86.67%)/6 (13.33%)	0.505
Physical activity level (low/Moderate/High), n (%)	22 (48.89%)/17 (37.78%)/6 (13.33%)	10 (22.22%)/25 (55.56%)/10 (22.22%)	0.021
Use of assistive device, n (%)	11 (24.44%)	0 (0.00%)	<0.001
fall history in past year, n (%)	14 (31.11%)	3 (6.67%)	0.003

KOA, knee osteoarthritis; SD, standard deviation; BMI, body mass index; n, number of participants; %, percentage; —, not applicable.

### Variables

#### Lower limb muscle strength assessment

Lower limb muscle strength was evaluated using a baseline handheld dynamometer (Lafayette Manual Muscle Tester, Model 01,165), a valid and reliable device for quantifying isometric force production in clinical populations, including those with musculoskeletal disorders (Hislop et al., 2022). Handheld dynamometry has demonstrated high intra- and inter-rater reliability, with intraclass correlation coefficient (ICC) values typically exceeding 0.85 for lower-limb muscle strength assessment in clinical populations (Lipovšek et al., 2022; Morin et al., 2023). The dynamometer was calibrated before each testing session according to the manufacturer’s instructions to ensure measurement accuracy. All measurements were conducted following standardized protocols with consistent examiner technique to ensure replicability and reduce inter-rater variability (Hislop et al., 2022). The dominant limb, identified as the leg used to kick a ball, was assessed for both quadriceps and hamstrings strength in a single session. In addition to the dominant limb, corresponding measurements were obtained for the contralateral limb using the same standardized protocol to calculate bilateral strength parameters, including the limb symmetry index. Before testing, participants received verbal instructions and a demonstration of the procedure, followed by a submaximal familiarization trial to ensure correct technique and minimize learning effects. Each contraction was sustained for 5 seconds, with standardized verbal encouragement (“Push as hard as you can and hold!”) provided throughout the test to promote maximal voluntary effort. A 30-s rest period was provided between trials to prevent fatigue.

For quadriceps strength, participants were seated upright on a flat plinth with hips and knees flexed to 90°, back supported, arms resting by their sides, and feet not in contact with the ground. The dynamometer was positioned just proximal to the lateral malleolus on the anterior aspect of the tibia. The examiner stabilized the limb by gently holding the posterior thigh to prevent hip movement. Participants were instructed to perform a maximal isometric knee extension against the device. Three trials were recorded, and the highest peak force (N) was retained for analysis. For hamstrings strength, participants remained in the same seated position. The dynamometer was repositioned to the posterior aspect of the tibia, just above the ankle joint. Participants were instructed to flex the knee maximally against the device, maintaining the contraction for 5 seconds while the examiner provided counter-pressure to ensure consistent resistance. The same three-trial procedure was followed, and the highest value was recorded.

The Q: H ratio was calculated by dividing peak quadriceps strength by the corresponding hamstring strength of the same limb, yielding a dimensionless measure reflecting relative muscle balance across the knee joint (Ruas et al., 2019). The Q: H ratio represents the relative strength balance between the knee extensors and flexors and is commonly used as an indicator of joint stability and neuromuscular coordination (Harput et al., 2014). This ratio has been used to assess neuromuscular coordination and joint stability, with lower values previously associated with altered joint mechanics and increased functional limitations in knee osteoarthritis (Costantino et al., 2025). Normative values for the quadriceps-to-hamstrings ratio typically range between 1.5 and 2.0 in healthy adults, and deviations from this range have been associated with impaired knee joint stability and altered biomechanics (Martín del Campo et al., 2025). Limb symmetry index (LSI) was calculated to assess strength balance between limbs (Kour et al., 2021). It was derived using the formula: (strength of the weaker limb/strength of the stronger limb) × 100 and expressed as a percentage (Mirković et al., 2022). An LSI value below 90% was considered indicative of clinically relevant asymmetry. Strength values from quadriceps testing were used for this calculation. This cutoff is consistent with previous literature, where an LSI value below 90% has been widely used to define clinically meaningful inter-limb asymmetry in musculoskeletal and rehabilitation research (Mirković et al., 2022). Quadriceps strength values from both limbs were used to derive the limb symmetry index by comparing the stronger and weaker limbs, ensuring that bilateral assessment procedures were applied consistently across all participants.

#### Postural stability assessment

**FIGURE 1:**
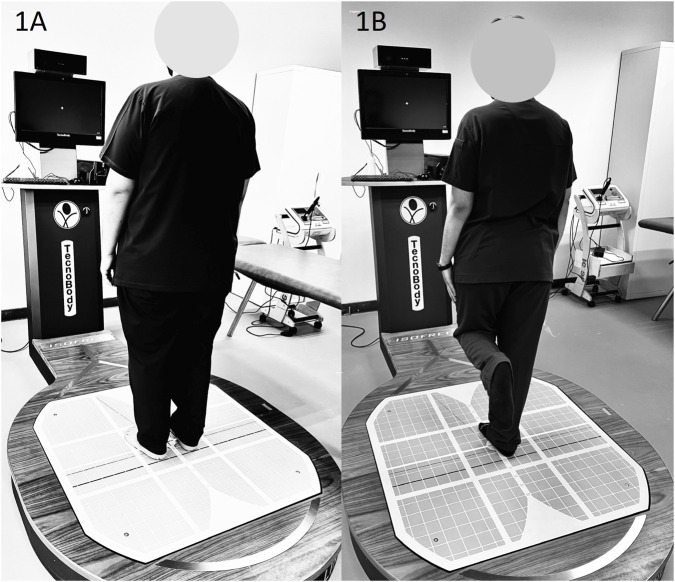
Posturography Assessment of Static Balance in Individuals with Knee Osteoarthritis. **(A)** Bipedal stance on a TecnoBody stabilometric platform under eyes-open condition. **(B)** Single-leg stance test performed on the same platform to evaluate unilateral balance and postural control.

Postural control was assessed using a static posturography system (Iso-free, Techno Body, Italy) (Raizah et al., 2023), which quantifies center-of-pressure (COP) displacement during quiet standing. Static posturography systems have also demonstrated excellent reliability, with reported intra- and inter-rater ICC values generally above 0.80 for COP based sway measures under both eyes-open and eyes-closed conditions (Yağcı et al., 2025). COP refers to the point location of the vertical ground reaction force vector and is commonly used to quantify postural sway during balance assessment (Shim et al., 2022). All participants were evaluated barefoot to minimize footwear variability and were instructed to stand on the force platform with feet shoulder-width apart and arms relaxed at their sides (Figure 1A). The head was maintained in a neutral position with gaze directed forward at a visual target placed at eye level, 2 m away. Each trial lasted 30 s, and participants were asked to “stand as still as possible without moving.” To ensure safety and minimize variability, testing was conducted in a quiet, well-lit environment under the supervision of a trained examiner for each trial. A 5-s stabilization period preceded each trial to allow the participant to attain a steady posture before data acquisition. A familiarization trial was provided before data collection. The dominant leg was not specifically weighted during the double-leg stance, and all assessments were performed under eyes-open and eyes-closed conditions in randomized order. Two trials were completed for each condition, with 60 s of rest between trials, and the average of both trials was used for analysis. The order of testing (eyes open or eyes closed) was randomized using a computer-generated sequence to minimize order effects.

The sway area was calculated as the 95% confidence ellipse encompassing the COP trajectory during the 30-s standing trial. The variable was recorded in square centimeters (cm^2^) and reflects the spatial dispersion of the COP. A larger sway area indicates greater postural instability. Eyes-open and eyes-closed conditions were tested separately to examine the influence of visual feedback on postural control. From the same posturography trials, anteroposterior (AP) and mediolateral (ML) sway ranges were derived as the maximum displacements of the COP in the sagittal and frontal planes, respectively. These values were expressed in millimeters (mm) and represent the amplitude of postural excursions in each direction. Larger sway ranges reflect reduced directional stability and are commonly linked with proprioceptive deficits and joint instability in individuals with OA (Zeng et al., 2022). The average of two trials per condition was used for statistical analysis. The system automatically computed sway velocity as the total COP path length divided by trial duration (30 s), reported in millimeters per second (mm/s). This variable captures the speed of postural adjustments and is considered a sensitive indicator of postural control efficiency. Both eyes-open and eyes-closed conditions were assessed, with higher velocities indicating poorer neuromuscular control and increased balance demands (Raizah et al., 2023).

#### Single-leg stance time

Single-leg stance time was evaluated using a digital stopwatch, with the participant instructed to stand on the dominant leg and lift the contralateral foot off the ground (Figure 1B). The dominant leg was defined as the leg used to kick a ball, consistent with standard limb dominance assessment protocols (Yokota et al., 2023). Arms were maintained at the sides, and vision was directed forward. Timing began when the non-stance foot was lifted and ended when the stance foot touched the ground, shifted, or reached the 30-s cutoff (Yokota et al., 2023). Verbal instructions were standardized: “When I say start, lift your foot and try to balance as long as you can without moving your other foot or hopping.” Three trials were completed with 30-s rest intervals, and the longest time (in seconds) was recorded for analysis. A stable chair was positioned nearby for safety, but was not used unless a loss of balance occurred.

Strength and balance assessments were conducted on the same day, with a 10-min rest period between sessions to minimize fatigue. The order of testing (strength vs. balance) was counterbalanced across participants.

#### Sample size calculation

The sample size was determined using *G*Power* version 3.1.9.7 based on a two-tailed Pearson correlation analysis, targeting an expected moderate effect size (r = 0.4) between quadriceps strength and postural sway, as reported in similar populations with knee osteoarthritis. Assuming an alpha level of 0.05, a statistical power of 0.80, and a 1:1 allocation ratio between the osteoarthritis and control groups, the required minimum total sample size was calculated to be 84 participants. To account for potential data loss or exclusions, the final sample size was set at 90 participants, with 45 per group.

### Data analysis

All statistical analyses were conducted using IBM SPSS Statistics version 24.0 (IBM Corp., Armonk, NY, USA). Before analysis, the data were screened for completeness and assessed for normality using the Shapiro–Wilk test and visual inspection of histograms and Q–Q plots, confirming that all continuous variables were normally distributed. Accordingly, parametric statistical methods were applied. Descriptive statistics, including means and standard deviations for continuous variables and frequencies with percentages for categorical variables, were used to summarize demographic and clinical characteristics. Group comparisons between individuals with knee osteoarthritis and controls were performed using independent samples t-tests for continuous variables and chi-square tests for categorical variables. Pearson’s correlation coefficients were calculated to assess the strength and direction of associations between lower limb muscle strength parameters and postural sway outcomes within the KOA group. To identify independent predictors of postural sway (sway area in the eyes-closed condition), a multiple linear regression analysis was conducted, entering quadriceps strength, hamstrings strength, age, body mass index, and limb symmetry index as predictor variables. Additional multiple linear regression analyses were conducted for sway area under eyes-open conditions, sway velocity, and single-leg stance time to provide a more comprehensive evaluation of determinants of postural control. Standardized and unstandardized beta coefficients, confidence intervals, and significance values were reported. A two-tailed p-value of less than 0.05 was considered statistically significant for all analyses. Assumptions of regression were assessed prior to model estimation. Homogeneity of variance was evaluated using Levene’s test, and multicollinearity was examined using variance inflation factor (VIF) values, all of which were within acceptable thresholds.

## Results

Individuals with knee osteoarthritis demonstrated significantly impaired postural stability across all measured sway parameters compared to controls, as shown in Table 2. Sway area was greater in both eyes-open (2.85 ± 0.73 vs. 1.92 ± 0.58 cm^2^, p < 0.001) and eyes-closed conditions (3.41 ± 0.89 vs. 2.25 ± 0.66 cm^2^, p < 0.001), with large effect sizes (Cohen’s d = 1.41 and 1.47, respectively). Similarly, anteroposterior and mediolateral sway ranges were significantly elevated in the KOA group (17.64 ± 3.21 mm and 14.38 ± 2.79 mm) compared to controls (13.12 ± 2.87 mm and 10.95 ± 2.46 mm), with mean differences exceeding 3.4 mm and Cohen’s d values above 1.3. Sway velocity was also markedly higher under both visual conditions, and single-leg stance time was reduced in KOA participants (8.42 ± 2.55 vs. 12.31 ± 2.84 s, p < 0.001), indicating compromised dynamic balance control.

**TABLE 2:** Comparison of postural sway parameters between KOA and control groups.

Sway parameter	KOA group (mean ± SD)	Control group (mean ± SD)	Mean difference [95% CI]	Cohen’s d	p-value
Sway area eyes open (cm^2^)	2.85 ± 0.73	1.92 ± 0.58	0.93 [0.65–1.21]	1.41	<0.001
Sway area eyes closed (cm^2^)	3.41 ± 0.89	2.25 ± 0.66	1.16 [0.83–1.49]	1.47	<0.001
AP sway range (mm)	17.64 ± 3.21	13.12 ± 2.87	4.52 [3.27–5.77]	1.48	<0.001
ML sway range (mm)	14.38 ± 2.79	10.95 ± 2.46	3.43 [2.29–4.57]	1.31	<0.001
Sway velocity eyes open (mm/s)	11.27 ± 2.14	8.49 ± 1.75	2.78 [1.92–3.64]	1.40	<0.001
Sway velocity eyes closed (mm/s)	13.05 ± 2.33	9.72 ± 1.91	3.33 [2.32–4.34]	1.54	<0.001
Single-leg stance time (s)	8.42 ± 2.55	12.31 ± 2.84	−3.89 [-5.08 to −2.70]	1.45	<0.001

KOA, knee osteoarthritis; SD, standard deviation; CI, confidence interval; cm^2^, square centimeters; mm, millimeters; mm/s, millimeters per second; AP, anteroposterior; ML, mediolateral; s, seconds.

Marked reductions in lower limb muscle strength were evident in individuals with knee osteoarthritis compared to controls, with the most pronounced deficit observed in quadriceps strength (232.65 ± 42.18 vs. 286.32 ± 38.49 N, mean difference −53.67 N, 95% CI: −69.88 to −37.46, p < 0.001; Cohen’s d = 1.32) (Table 3). Hamstring strength was also significantly lower in the KOA group (142.88 ± 31.15 vs. 165.79 ± 29.46 N, p < 0.001), and the quadriceps-to-hamstrings ratio showed a modest reduction (1.63 ± 0.28 vs. 1.75 ± 0.25, p = 0.021). Limb symmetry index was substantially reduced in KOA participants (81.74% ± 7.22% vs. 95.13% ± 5.88%, p < 0.001), reflecting notable bilateral strength imbalance with a large effect size (Cohen’s d = 2.06).

**TABLE 3:** Comparison of lower limb muscle strength between KOA and control groups.

Muscle strength parameter	KOA group (mean ± SD)	Control group (mean ± SD)	Mean difference [95% CI]	Cohen’s d	p-value
Quadriceps strength (N)	232.65 ± 42.18	286.32 ± 38.49	−53.67 [-69.88 to −37.46]	1.32	<0.001
Hamstrings strength (N)	142.88 ± 31.15	165.79 ± 29.46	−22.91 [-35.08 to −10.74]	0.74	<0.001
Quadriceps: Hamstrings ratio	1.63 ± 0.28	1.75 ± 0.25	−0.12 [-0.22 to −0.02]	0.46	0.021
Limb symmetry index (%)	81.74 ± 7.22	95.13 ± 5.88	−13.39 [-16.35 to −10.43]	2.06	<0.001

KOA, knee osteoarthritis; SD, standard deviation; CI, confidence interval; N, newtons; %, percentage.

**FIGURE 2:**
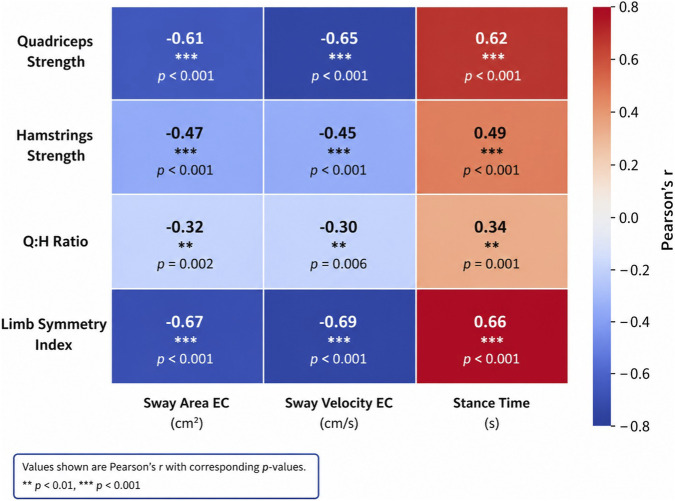
Heatmap of Pearson correlation coefficients between lower limb muscle strength parameters and postural sway measures in individuals with knee osteoarthritis.

Significant correlations were found between lower limb muscle strength and postural sway parameters in the KOA group, with stronger muscles generally associated with better postural control (Figure 2). Quadriceps strength showed moderate to strong negative correlations with sway area (r = −0.61) and sway velocity (r = −0.65) in the eyes-closed condition, and a positive correlation with single-leg stance time (r = 0.62), all with p < 0.001. Hamstrings strength demonstrated weaker but still significant associations with sway metrics (r range: −0.42 to −0.49, p = 0.002). The quadriceps-to-hamstrings ratio was modestly correlated with sway outcomes (r = −0.30 to 0.34, p = 0.027). The limb symmetry index showed the strongest overall correlations, particularly with sway velocity (r = −0.69) and stance time (r = 0.66), highlighting the functional impact of bilateral strength imbalance.

**FIGURE 3:**
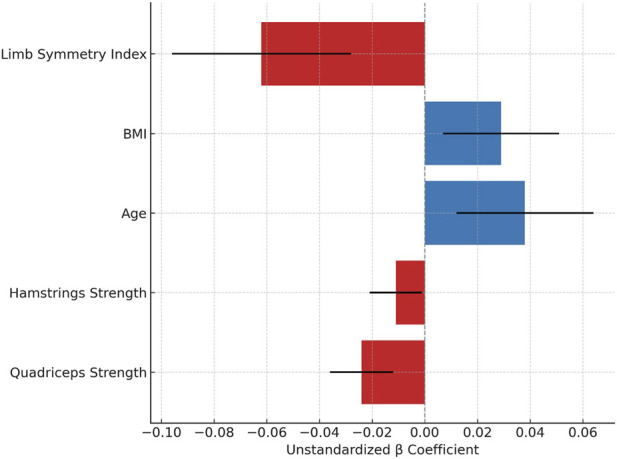
Unstandardized β coefficients and 95% confidence intervals for predictors of sway area (eyes closed) in individuals with knee osteoarthritis.

In the multiple linear regression analysis, lower quadriceps strength emerged as the strongest independent predictor of increased sway area in the eyes-closed condition among individuals with KOA (β = −0.024, 95% CI: −0.036 to −0.012, standardized β = −0.41, p < 0.001) (Table 4; Figure 3). Hamstring strength was also a significant predictor, though with a smaller effect size (β = −0.011, p = 0.037). Older age (β = 0.038, p = 0.005) and higher BMI (β = 0.029, p = 0.011) were positively associated with greater postural sway, indicating worse stability. Limb symmetry index demonstrated a meaningful inverse relationship with sway area (β = −0.062, p = 0.001), suggesting that greater inter-limb strength asymmetry contributes to postural instability.

**TABLE 4:** Multiple linear regression–predictors of postural sway (sway area eyes closed) in KOA group.

Predictor variable	Unstandardized β	Standard error	95% CI	Standardized β	p-value
Quadriceps strength (N)	−0.024	0.006	−0.036 to −0.012	−0.41	<0.001
Hamstrings strength (N)	−0.011	0.005	−0.021 to −0.001	−0.25	0.037
Age (years)	0.038	0.013	0.012 to 0.064	0.31	0.005
BMI (kg/m^2^)	0.029	0.011	0.007 to 0.051	0.28	0.011
Limb symmetry index (%)	−0.062	0.017	−0.096 to −0.028	−0.36	0.001

KOA, Knee Osteoarthritis; β, beta coefficient; CI, Confidence Interval; N, Newtons; BMI, Body Mass Index.

## Discussion

This study aimed to examine the relationship between lower-limb muscle strength and postural stability in individuals with knee osteoarthritis, using objective assessments of sway parameters and isometric strength. The findings demonstrated that individuals with osteoarthritis exhibited significant impairments in postural control compared to matched controls, characterized by greater sway magnitudes and reduced single-leg stance time. Marked reductions in quadriceps and hamstrings strength, altered strength ratios, and substantial inter-limb asymmetry accompanied these deficits. Furthermore, significant associations were identified between muscle strength variables and balance performance, with quadriceps strength and limb symmetry emerging as key contributors to postural stability. Collectively, the results underscore the functional relevance of neuromuscular deficits in individuals with knee osteoarthritis and support the integration of strength-based metrics in postural assessments. While previous investigations have examined lower-limb muscle strength or postural stability in knee osteoarthritis separately, and some have reported associations between these variables, the present study makes several novel contributions. First, it integrates objective, device-based isometric dynamometry and quantitative static posturography into a single clinically feasible assessment protocol for routine rehabilitation settings. Second, beyond evaluating absolute quadriceps and hamstrings strength, this study systematically incorporated the quadriceps-to-hamstrings ratio and the limb symmetry index, enabling a more comprehensive biomechanical characterization of inter-limb imbalance. Third, using multiple linear regression, the study identified quadriceps strength and limb symmetry as independent predictors of sway area under eyes-closed conditions, thereby isolating their relative contributions while controlling for age and body mass index. This combined analytical and methodological approach advances current understanding by clarifying not only the presence of strength and balance deficits in knee osteoarthritis, but also their hierarchical and clinically actionable relationships.

The observed impairments in postural stability among individuals with knee osteoarthritis can be attributed to a combination of altered joint biomechanics, proprioceptive deficits, and neuromuscular dysfunction commonly associated with the disease (Zeng et al., 2022; Ganjeh et al., 2025; Reddy et al., 2023a; Alshahrani and Reddy, 2025; Alshehri et al., 2025a; Alshehri et al., 2025b; Reddy et al., 2023b; Alahmari and Reddy, 2024a; Kardm et al., 2025; Alfaya et al., 2023). Structural changes in the knee joint, including cartilage degradation and osteophyte formation, disrupt afferent sensory input and impair the ability to maintain stable postural control, particularly under challenging conditions such as an eyes-closed stance (Liu et al., 2025). Several studies have similarly reported increased postural sway in individuals with knee osteoarthritis compared to healthy controls, confirming the robustness of these findings (Labanca et al., 2021). Labanca et al. (Labanca et al., 2021) demonstrated increased postural sway and reduced proprioceptive acuity in knee OA, linking joint damage to impaired stability. Sabashi et al. (2021) also identified significant differences in static and dynamic balance between OA patients and healthy adults, reinforcing the role of joint pathology in postural control deficits. Zeng et al. (2022) reported that altered sensorimotor function and knee pain were associated with postural instability, particularly in single-leg stance. Additionally, Lee et al. (2022) found that increased sway in OA patients was independent of pain severity, suggesting that joint structural changes and neuromuscular factors are dominant contributors to balance impairments. These consistent findings across the literature align with the current study’s results, confirming that postural sway is significantly elevated in knee osteoarthritis across multiple conditions and metrics.

The associations identified between lower limb muscle strength and postural stability in individuals with knee osteoarthritis are likely due to direct biomechanical dependencies between muscular force generation and balance control (Zeng et al., 2022; Da et al., 2024; Alahmari and Reddy, 2024b). From a mechanistic perspective, quadriceps weakness and inter-limb asymmetry contribute to impaired proprioceptive feedback and joint position sense, which are critical for maintaining postural equilibrium (Koura et al., 2026). In knee osteoarthritis, degeneration of articular cartilage and associated mechanoreceptors reduces afferent input from the joint, thereby diminishing sensorimotor integration (Marks, 2024). This disruption compromises the central nervous system’s ability to accurately detect joint position and movement, resulting in delayed or inappropriate postural responses (Marks, 2024). Furthermore, altered joint biomechanics, including reduced knee extensor moment and compensatory movement strategies, increase reliance on passive structures and non-optimal muscle activation patterns (Salamanna et al., 2023). Inter-limb asymmetry exacerbates these effects by creating unequal load distribution and instability during weight-bearing tasks, thereby increasing center-of-pressure displacement and postural sway (Salamanna et al., 2023). Neuromuscular control is further impaired by arthrogenic muscle inhibition, which limits effective quadriceps activation and reduces the capacity to generate timely stabilizing forces (Salamanna et al., 2023).

Quadriceps strength, in particular, plays a central role in maintaining knee joint stability and supporting the body’s center of mass during static and dynamic tasks (Alshahrani and Reddy, 2023). Several studies have established a clear relationship between diminished lower limb strength and impaired balance. Hislop et al. (Hislop et al., 2022) demonstrated that reduced quadriceps strength was associated with poorer balance performance and increased fall risk in older adults with knee OA. Chaharmahalil et al. (Chaharmahali et al., 2021) found that greater muscle strength predicted better postural control outcomes and reduced sway. Similarly, Jorge and Dionisio (2021) reported significant correlations between quadriceps strength and single-leg stance performance in patients with OA, highlighting its clinical relevance. In support of this, Alkhamis et al. (2024) observed that lower-limb strength asymmetry was associated with balance deficits, particularly in unilateral OA, underscoring the importance of limb symmetry for postural control. The current study confirms and extends these findings by demonstrating that not only absolute strength but also strength ratios and inter-limb symmetry are critical determinants of postural stability, as evidenced by the strong correlations and regression outcomes observed (Wang et al., 2025).

From a clinical perspective, these findings support the implementation of targeted rehabilitation strategies focusing on quadriceps strengthening and restoration of limb symmetry. Achieving a limb symmetry index of ≥90% should be considered a minimum functional goal to reduce asymmetry-related instability. Progressive resistance training programs that emphasize quadriceps activation—typically performed at 60%–80% of one-repetition maximum, 2–3 times per week for at least 8–12 weeks—may be effective in improving strength and postural control (Ahsan and Alzahrani, 2024). In addition, neuromuscular and proprioceptive training, including balance exercises under reduced visual input conditions, should be integrated to enhance sensorimotor function (Sluga and Kozinc, 2024). These combined approaches are likely to improve postural stability and reduce the risk of falls in individuals with knee osteoarthritis.

In addition to muscle strength and limb symmetry, body mass index emerged as an independent predictor of sway area under eyes-closed conditions in the regression model. This finding indicates that higher BMI contributes to impaired postural stability beyond the effects of neuromuscular deficits related to knee osteoarthritis (Zeng et al., 2022). Increased body mass may alter center-of-mass dynamics, joint loading, and sensorimotor control, thereby exacerbating balance impairments (Zeng et al., 2022). Clinically, these results highlight the importance of considering weight management strategies alongside strength and balance rehabilitation to optimize postural stability in individuals with knee osteoarthritis.

### Clinical significance

The findings of this study highlight the critical role of lower limb muscle strength, particularly quadriceps force and inter-limb strength symmetry, in maintaining postural stability in individuals with knee osteoarthritis. The use of clinically accessible tools, such as posturography and handheld dynamometry, enabled the objective identification of balance impairments and neuromuscular deficits in this population. These results emphasize the importance of integrating quantitative assessments of strength and balance into routine clinical evaluations for knee osteoarthritis. Furthermore, the demonstrated associations support prioritizing targeted strength training and symmetry restoration in rehabilitation protocols to reduce postural instability and fall risk.

### Limitations of the study and areas of future research

This study has several limitations that should be considered when interpreting the findings. First, its cross-sectional design precludes causal inference about the relationship between lower-limb muscle strength and postural control in individuals with knee osteoarthritis. Second, the sample consisted of community-dwelling older adults with moderate disease severity (Kellgren–Lawrence grades II–III), which may limit the generalizability of the results to individuals with advanced osteoarthritis or those in institutional or inpatient settings.

Body composition was not assessed, representing an additional methodological limitation. Measures such as total lean mass, segmental muscle mass, and body fat percentage were not obtained, although these factors influence muscle force production and postural control. Consequently, the observed reductions in quadriceps and hamstrings strength cannot be attributed solely to neuromuscular impairment or disease-related mechanisms. Pain intensity was not quantified using standardized instruments such as the VAS, which is a relevant confounding variable. Pain may directly influence muscle activation, strength performance, and balance control, and its omission limits the ability to fully interpret the interaction between symptoms and functional outcomes. Although objective strength and balance assessments were conducted, dynamic gait analysis was not included. This limits the evaluation of postural control during functional activities such as walking, where instability may be more pronounced. Additionally, no subgroup analysis was performed to distinguish between unilateral and bilateral knee osteoarthritis, which may exhibit different biomechanical adaptations, patterns of asymmetry, and functional impairments. Furthermore, although key covariates such as age and body mass index were included in the regression model, other factors such as physical activity level and fall history were not incorporated, as they may represent downstream behavioral consequences of the condition rather than independent physiological predictors. However, their exclusion may still limit comprehensive modeling of balance performance.

Future research should adopt longitudinal and interventional designs to establish causal relationships and evaluate the effectiveness of targeted rehabilitation strategies. Incorporating comprehensive pain assessment, objective body composition analysis, and dynamic gait evaluation would enhance mechanistic understanding. In addition, subgroup analyses based on disease laterality and severity, along with the integration of wearable sensor technologies for real-world monitoring, may provide deeper insight into functional impairments and support the development of individualized rehabilitation protocols.

## Conclusion

This study demonstrated that individuals with knee osteoarthritis exhibit significantly impaired postural stability and reduced lower-limb muscle strength compared with healthy controls. Quantitative analysis revealed that quadriceps strength and limb symmetry are key contributors to balance performance, with strong correlations between strength variables and sway parameters. These findings reinforce the relevance of muscle strength in postural control and support the use of simple, objective tools to guide assessment and rehabilitation in individuals with knee osteoarthritis.

## Data Availability

The datasets presented in this study can be found in online repositories. The names of the repository/repositories and accession number(s) can be found in the article/supplementary material.
